# Some Recent Advances in Ophthalmology
*A paper read before the Bristol Medico-Chirurgical Society, 8th March, 1939.


**Published:** 1939

**Authors:** Anthony Palin

**Affiliations:** Honorary Assistant Surgeon, Bristol Eye Hospital; Senior Clinical Assistant, Royal Westminster Ophthalmic Hospital, London


### The Bristol Medico-Chirurgical Journal

*" Scire est nescire, nisi id me
Scire alius sciret."*

SUMMER, 1939.

### SOME RECENT ADVANCES IN OPHTHALMOLOGY.*

BY

ANTHONY PALIN, B.M., B.Ch., F.R.C.S.,

*Honorary Assistant Surgeon, Bristol Eye Hospital; Senior Clinical Assistant, Royal Westminster Ophthalmic Hospital, London.*

### CONTACT LENSES.

*History and mode of use.*

Although it is only within very recent years that corneal contact glasses have become a practical proposition, the idea is by no means new.

As so few people have had an opportunity of seeing a contact lens, I should explain that this term refers to glass lenses which are worn inside the eyelids in direct contact with the eyeball itself. They are invisible to ordinary observation and indeed even to

* A paper read before the Bristol Medico-Chirurgical Society, 8th March, 1939.

H

close inspection, if it is not known that they are present. (Before a recent lecture to the nurses at the Eye Hospital I inserted contact lenses into my own eyes and let them each examine me, merely mentioning that there was something unusual about my eyes. Not one nurse realized that I was wearing contact glasses. And I once asked a London ophthalmic surgeon, well known as the author of a text-book, to examine a patient who was fitted with them. He completely failed to note that he had been examining the eyes through contact lenses.)

To revert to their historical aspect, it is interesting to find that in 1827 Sir John Herschel, brother of the Astronomer Royal, conceived the idea of using such lenses, although he was unable to carry it out. The first lens was made by Fick in 1887, and during subsequent years a few lenses, blown from molten glass, were made by Müller in Berlin ; while at the same time Sultzer was working on contact lenses ground from solid blocks of glass. Nevertheless no really satisfactory clinical results could be obtained, and no further progress was made.

More than one hundred years after Herschel an important milestone has been passed, and now contact lenses can be obtained in stock sizes, or made to measure. Their prescription and fitting are matters of extreme precision and, in the case of those made to measure, involve a mould being taken of the eye and much time to ensure the accurate fit which is essential for comfort and efficiency.

Each lens is a half to one millimetre thick and has two segments—a central domed portion which lies next to the cornea itself, and a peripheral portion adjusted to the radius of curvature of the sclera. Any gap or irregularity between the front of the cornea and back of the lens is occupied by normal saline. This gives an artificial anterior chamber of almost equal refractive index to that of the anterior part of an eyeball; so that the refraction of all visual rays now takes place at the smooth anterior surface of the contact lens and not at the cornea.*

Insertion and removal of the glass is achieved by means of a special rubber sucker, or in some cases direct by the fingers. At the first trials, to give confidence and prevent blinking, it is usually necessary to anæsthetize the eye with holocaine or cocaine.

There are two distinct sets of cases in which this type of glass is used. In the first it functions as a protective agent and in the second it is used for optical effects.

A. *As a protective medium.*

In this capacity the contact glass has at present small but valuable scope. One may sub-divide this group of patients into: (i) Those who use contact glasses to avoid certain hazards, and (ii) Those whose corneae need protection for pathological reasons.

(i). The former class is comprised of those whose occupations or pastimes may involve accidents in which ordinary spectacles might be broken. Such are, to mention a few, airmen, racing-drivers, footballers, skiers and those who shoot or hunt.

One may digress for a moment here. It is not perhaps easy to realize that a glass in contact with the eyeball is, if broken, less likely to cut the eye than the glass in an ordinary spectacle frame. Sir Stewart Duke-Elder has ascertained that this is the case, and I have devised an experiment to prove it.

* In the case of the glasses made to measure there is no artificial anterior chamber, there being complete capillary cohesion between the cornea and the inside of the contact lens.

A sheet of rubber is fixed in a box a short distance behind a sheet of glass. Breaking the glass with a hammer results in several fragments of glass being driven through the rubber. In the second set of conditions the glass and rubber are in contact. In the latter case breaking the glass results in fragmentation but no particles pierce the rubber.

An example of protection occurred quite recently when a motorist involved in a crash had his eyelids badly cut by broken windscreen glass. By virtue of the contact lens he was wearing, however, the eyeball completely escaped injury.

(ii). The class of patient whose cornea needs protection for pathological reasons mainly comprises those who have intractable corneal conditions, such as relapsing keratitis—especially of the neuroparalytic type. This includes herpes ophthalmicus, cases of facial neuralgia which have had Gasserian root section or alcohol injection, whose corneal sensitivity does not return, conjunctival pemphigus, and some cases of old keratitis from mustard gas. These cases present a very real problem, as those of you who have had the care of such patients will know. The cumbersome protective goggle which is so often necessary can be replaced by a much neater article in the form of a contact glass.

Surgical ophthalmology has also found a use for these lenses in applying them after corneal grafting operations to maintain the transplant in correct position in the corneal bed. This method, however, is less generally adopted by ophthalmic surgeons than the use of overlying sutures or a conjunctival flap.

An interesting protective use exists for those rare individuals of marked albino type, and those with partial or total congenital absence of iris, where a constant source of misery is photophobia, due to lack of uveal pigment. This is dealt with by having the central or peri-central portion of the contact glass tinted sufficiently to cut down the admitted light to the individual's tolerance point.

Mention of tinted contact lenses reminds me of a novel use to which one was recently put in America. A wanted criminal, after much effort on the part of the police, was at last — they thought — apprehended. However, the criminal's dossier showed that he had blue eyes. This man had brown eyes, and, after police and medical examinations, was about to be released when suspicion became so strong that his eyes were re-examined by an ophthalmic surgeon. It was then found that he was wearing brown-tinted contact lenses ! The gaol kept its prisoner and the Police Museum acquired an unusual exhibit.

B. *Use for optical effects.*

The outstanding success of the contact lens is in keratoconus (or conical cornea). In this condition there is a degenerative thinning of the centre of the cornea which causes a bulge, and a degree of irregular astigmatism which cannot be corrected with ordinary glasses and which produces distortion in the whole field of vision. This means that in many such cases the top letter of the ordinary Snellen test-type cannot be read at three yards even with the best glasses. A contact glass will often allow all the letters on the chart to be read at the full distance of twenty feet.

Myopia is another type of refractive error in which sufferers may be grateful for a contact glass, although vanity is often the urge which leads to them. Actresses, for example, whose "appeal" would be reduced by wearing glasses, and who would blunder about on the stage without them, are glad of this invisible aid to perfect vision. And I know of a very short-sighted woman, who, about to be presented at Court, could not suffer to reveal to the general gaze her much-photographed face in the powerful lenses she needed to turn her beautiful but vacant gaze into an accurate regard. She obtained contact lenses for fear of tripping over the edge of the royal carpet. Apparently, however, she wore them on that occasion only. Vanity aside, the high myope may find contact glasses of very special help, for example when bathing, as they are no more affected than the eyeball by drops of water. The short-sighted surgeon also (who, one hopes, is not actuated by considerations of vanity) may find them of great advantage in operating, since these lenses do not become steamy.

Unilateral aphakia due to removal or traumatic destruction of one lens, usually leads to the affected individual not being able to see accurately with both eyes at the same time. The reason for this is that the powerful convex lens which he now requires, and which in a spectacle frame is ordinarily about twenty millimetres in front of the optical centre of the eye, produces marked magnification in that eye and thus gives diplopia. The contact glass, worn so close to the optical centre, does not give any magnification and allows the individual once again to have accurate binocular vision.

*Disadvantages.*

It must not be thought that contact glasses are Utopian in character. Unfortunately there has recently been a spate of publicity, not only in the more sensational papers but also in quieter periodicals, and broadcasts, too, have made a contribution. Like other news-value subjects such as improving the quality of cock's combs and bringing down testicles, contact lens potentialities have brought innumerable requests for treatment from totally unsuitable cases. The benefit to the individual concerned must be so considerable as to render trivial certain drawbacks which would trouble most of us.

The average person does not find the lenses easy to insert and if an air-bubble is left between the cornea and the lens, the latter might quite likely drop out, and further, the correct optical effect is abolished. Cocaine or holocaine has to be used until the patient gets used to the new sensation, and in some cases even after that, for the lens is undoubtedly a conjunctival foreign body. Many patients can never get accustomed to the lenses and even among regular wearers tolerance to wear varies from two to twelve hours, at the end of which time the lenses must be removed and washed and the eyes allowed a little time to recover before reinsertion.

Not the smallest question is that of expense. The ready-made contact lenses of Zeiss cost five pounds per lens. The made-to-measure ones need so many fittings to grind to the correct thousandth of an inch that they may ultimately cost as much as forty-five pounds a pair. They can be insured, however, and in any case they will not break unless they are dropped on a very hard surface.

In spite of the drawbacks contact glasses will increase in use, although of course they are never likely to become of universal application. The big points in their favour are that unlike ordinary glasses they do not at all restrict the field of vision and they move with the eyeball. Furthermore, being so close to the eye they produce no distortion, and in selected cases give a degree of visual improvement unobtainable in any other way.

### DETACHMENT OF THE RETINA.

This condition is well worthy of consideration under the heading of Recent Advances, since a new treatment has entirely revolutionized the outlook. There are two main groups of detachment cases ; first, the exudative type, associated with diseases such as syphilis, tubercle, nephritis, venous thrombosis and neoplasm ; and secondly, the non-exudative type, due to inflammatory and degenerative conditions in the choroid such as myopia, and direct or indirect trauma. There are also a few cases in this group which fall under the designation "idiopathic." The treatment of the first group is essentially medical, except obviously in the case of neoplasm, where excision of the eye is indicated.

It is the second group that I want to discuss, for here a new surgical technique has turned a virtually untreatable disease (the failures formerly amounted to 98 per cent.) into one with a reasonable prospect of cure. In competent hands about 30 to 40 per cent. of these cases may now expect a good result.

### *Investigation and diagnosis*

It is important that every case of detachment be treated as a real emergency, since every hour that the patient spends out of bed after the onset increases the detachment and reduces the chances of cure. A review of signs and symptoms may assist in early recognition.

The first symptom is nearly always a major degree of loss of sight, although this may have been preceded by the sensation of flashes of light or streaks. The patient may also complain of "a curtain coming across the eye." The differential diagnosis of sudden loss of sight is fairly simple. Having excluded hysteria and long-standing blindness in one eye (which may only be suddenly discovered by accidental covering of the good eye) there are three painless causes of sudden blindness and one cause with pain.

The painless causes are :—

(i). Vascular accident, usually retinal hæmorrhage, embolism or thrombosis and (rarely) spasm of retinal arteries. Occasionally a retro-ocular or intra-cranial hæmorrhage may have to be excluded, though this is generally easy on account of other signs ;

(ii). Acute retrobulbar neuritis, with its characteristic pupil reaction, and pain on movement of or pressure on the eyeball ; and

(iii). Retinal detachment.

The painful cause is, of course, acute glaucoma, which should be obvious on account of the raised tension, hazy cornea, semi-dilated inactive pupil, and, usually, general prostration due to the severe pain.

As regards the signs of detachment, it is generally possible to see with the ophthalmoscope, preferably through the dilated pupil, a greyish area of retina which lies in corrugated folds and appears to balloon forwards, requiring a stronger plus lens to focus it than is required by the adjacent retina. The arteries and veins are markedly tortuous, darker in colour than normal, and appear to be contracted. The other classical sign is loss of an area of the visual field which is easily charted out on the perimeter.

Thorough examination makes a big difference to the technique decided upon and to the prognosis. The possibility of a neoplasm (which is usually a melano-sarcoma of the choroid) must be eliminated, and its absence is suggested by negative trans-illumination, normal intra-ocular tension, the presence of a retinal hole and the history of onset. One must also make certain that the condition is not part of a metabolic or vascular exudation.

The tedious part of the investigation is the search for a retinal hole or tear, for unless this is closed, fluid will again seep through between the choroid and retina and cause a recurrence. Dark-room inspection under full dilatation of the pupil is carried out with a focussed beam from the ophthalmoscope, and particular attention is paid to the periphery of the retina near its forward attachment to the ciliary body, since the majority of holes occur nearer the anterior than the posterior limits of the retina. When no hole or tear of the retina from its insertion can be found, puncture of the sclera with withdrawal of sub-retinal fluid may allow the retina to sink back on to the choroid and reveal one or the other.

*Technique of treatment.*

The principle of modern treatment is to drain off the sub-retinal fluid and to produce an aseptic reaction in the sclera, choroid and retina by carefully graded surgical diathermy, in such a way that when the retina falls back into place all three layers will adhere together. The area of detachment is marked out on a chart and the sites of any holes or tears are recorded to the precise millimetre. Under retro-bulbar novocaine the required area of sclera is bared, and muscles retracted or temporarily divided if necessary. With the aid of the chart the hole is marked on the outside of the sclera with methyl violet, and this area is then treated by twenty to sixty diathermy applications by special applicators, some of which make minute perforations in the sclera. A current of forty to eighty milliampères is used for three to eight seconds at each application.

When the sclera has been treated to the exact degree required over the appropriate area, the globe is punctured and the sub-retinal fluid allowed to drain off. This is usually done by cautery puncture or by trephining. The situation and effect of the process may be checked throughout by the ophthalmoscope, the treated areas appearing as dead-white patches in the retina.

After closure of the wound the patient is put straight back to bed and has to remain almost motionless and recumbent for upwards of three weeks. In satisfactory cases the whole retina becomes and remains firmly attached to the choroid and sclera, and normal vision is recovered over the full field. The long convalescence is tedious, but is well justified by the good results that one can now obtain.

### ORTHOPTICS IN THE TREATMENT OF SQUINT.

When about thirty-five years ago Claud Worth devised his first amblyoscope he had in mind many of those principles which to-day constitute the new science of Orthoptics.

Briefly to define orthoptic investigation and treatment, one may say that it is a branch of ophthalmology, devoted mainly to squint and partly to certain types of eye-strain, in which considerations of ocular function take precedence over all else. Until recent years, and even to-day in some quarters, the standard by which squint treatment has been judged has been a cosmetic one. If the eyes appeared straight, then both patient and ophthalmologist were satisfied. It was the tragedy of such a doctrine that the patient's normal appearance concealed from the world, and often from himself, the fact that he was usually almost blind in one eye and seldom had central binocular vision.

Such a person would lack all sense of stereoscopy, the most important faculty for judging distance and dimension. In growing children lack of central binocular vision has often been found to be the reason for their being hopelessly bad at games.

Many people ask one why an eye that has squinted becomes "lazy," as it is called by the laity. The mechanism is purely a compensatory one. An individual who has the visual axis of one eye deviated from normal must ordinarily suffer from diplopia. The effect of double vision is extremely uncomfortable and the brain in most cases suppresses the vision in the squinting eye. If this happens during the early years of life, when retinal perception is still being developed, the faculty of sight diminishes in the eye concerned. Thus it is that one so often finds in later years an eye with no discernible abnormality whose function and vision are defective. The object of orthoptics, like orthopædics, is primarily to restore function.

It must not be thought that this is a mode of treatment which replaces all existing procedures. What is claimed for it is that, after the correction of refractive errors (which is a preliminary in any case of squint) it will cure many cases functionally and cosmetically without operation. And in many other cases, by pre- and post-operative exercises, it will render an operative result more certain and permanent and will restore normal function.

In the past the average case of squint was left until puberty or later so that it could be operated on under local anæsthesia, which explains why one sees so many squinting adults and young people about to-day. The average ophthalmologist thus took little or no interest in function and contented himself with surgically adjusting the eyes as straightly as possible: and after the operation everyone would say it was a beautiful result. The important point is that not only might the vision in one eye remain grossly reduced—perhaps to 1/60—but also the two eyes were probably never used together. On the other hand, if the retina has been properly stimulated on orthoptic principles some of the vision will return (perhaps even all of it) and binocular function may be restored.

*Types of cases suitable.*

Several points have to be taken into consideration since many cases are quite unsuitable. For example, definite paralysis of one of the external ocular muscles will prevent useful results being obtained unless the paralysis is very slight or due to a recent injury.

Marked amblyopia presents a difficulty, unless it responds well to occlusion of the better eye. In general, occlusion should be carried out until the vision in the lazy eye is equal or nearly equal to that in the normal eye, before attempting orthoptic exercises.

In any case treatment is not feasible unless the squinting eye has, or can acquire, a vision of at least 6/18. Loss of central fixation is a contra-indication unless it is associated with what is called false projection. This is a condition resulting from long-standing deviation of an eye with development of a higher grade of vision in that area of retina immediately opposite the entering rays of light. The condition is sometimes badly described as "false macula." Lastly, it is no good for a child to start treatment unless it can come regularly for a minimum of two visits a week, and it must be at least four years old, unless it is highly intelligent.

*Principles of treatment.*

These vary somewhat in different cases. In the unilateral concomitant convergent type, which is by far the commonest, the first two stages are to correct any error of refraction (using a mydriatic) and to abolish or reduce any amblyopia by covering the good eye for two months or more. Subsequent stages —apart from operation if it should be necessary—are carried out with various orthoptic instruments which I will shortly describe. Most of them make use of pairs of lantern slides or illuminated pictures.

The third stage is abolition of false projection. Unless this is done, quite good function may be restored, yet the eye will remain deviated because it has accustomed itself to seeing best with an eccentric or non-macular area of the retina.

The fourth stage consists in the abolition of suppression with establishment of simultaneous perception, which is the lowest grade of binocular vision. The satisfactory attainment of this stage is marked by the patient having conscious diplopia, and, distressing as it may sound, this opens the gate to big advances.

The fifth stage is the development of the fusion sense. Here one wants to teach the patient to superimpose two images which, though similar, are not identical. The principle is to encourage the patient to see the complete picture. If he is suppressing vision in one eye, parts of the picture will fade out. This may be difficult or impossible in some patients suffering from a congenital defect of the fusion sense, presumably due to an error in their cortical control. In any case orthoptic treatment is the only method likely to re-establish it.

The sixth and last stage is the cultivation of stereoscopic vision, which is the highest grade of binocular vision.

If the patient can be successfully taken through all this there is a good chance, unless the squint is very marked, that he can be cured without operation. If he should require an operation, as for example in cases where there is a rigidly contracted muscle, the treatment will be of great value in giving him the necessary stimulus for binocular vision. This will make him keep his eyes straight and avoid that unfortunate sequel of so many squint operations—recurrence of deviation.

*Orthoptic treatment in non-squinting cases.*

In this highly civilized and somewhat neurotic age, ever-increasing numbers of adult patients present themselves with vague ocular symptoms, and are found to have little or no error of refraction. About a quarter of these cases have either :—

(i). An error consisting of external ocular muscle imbalance, or

(ii). An inadequacy of convergence amplitude for close vision.

In the first group the horizontal errors, *exophoria* (usually found in myopes), and *esophoria* (usually found in hypermetropes), are the most common and imply a tendency to convergent or divergent squint respectively ; but this is only revealed by some such test as covering the eyes with red and green glasses, or by using the Maddox rod, when the deviation at once becomes apparent. The vertical error, hyperphoria, and the torsional error, cyclophoria, although rarer, are more often the cause of symptoms. Hyperphoria and cyclophoria occasionally give rise to the interesting and often misdiagnosed condition of ocular torticollis, the characteristic feature of which is an upward deviation of the contra-lateral eye on looking to the side of the head-tilt. Unlike ordinary congenital torticollis, it generally causes the chin to be tilted down and turned to the same side. The mechanism is most frequently due to a congenital paresis of one superior rectus which results in overaction of its synergist in the other eye, the inferior oblique.

In the second group, inadequate convergence is often seen in elderly people, apparently associated with loss of accommodation ; but it is mainly between the ages of fifteen and thirty-five that symptoms occur, either as a congenital defect of binocular control or as the result of mental or physical fatigue states.

All these errors may usually be treated—and about 80 to 90 per cent. of them cured—by appropriate orthoptic exercises. It is of interest, too, that the Air Force ranks them as important as errors of refraction, for it is just these defects which may cause a pilot to make bad landings. Of them all, ocular torticollis is the least amenable and usually requires a myectomy of the overacting inferior oblique.

*Chief orthoptic instruments.*

*Synoptophore*—The most generally used muscle and fusion training apparatus, equipped with pairs of slides such as the lion and cage, the two rabbits (one with flowers and no tail, and the other with tail and no flowers) and ordinary stereoscopic pictures.

*Myoscope.*—A device for throwing two fusible pictures on a screen and giving exercising movements for any muscle desired.

*Cheiroscope.*—A drawing instrument to stimulate simultaneous perception, usable at home by intelligent children, in which a picture seen by one eye is, after relaying through the brain, projected by the other eye in such a way that the child is able to "trace" it on blank paper.

### Other Advances.

*Corneal Grafting.*—One of the most interesting of all advances is in the realm of corneal grafting, recently described to this Society by Mr. Tudor Thomas, of Cardiff.

In general the most satisfactory patients are those with grossly defective sight in both eyes, due to old interstitial keratitis or conditions such as ophthalmia neonatorum or burns which have produced widespread corneal opacity. During the past year this operation has been successfully performed on several occasions at the Bristol Eye Hospital.

*Colour Photography.*

Colour photography of the fundus with the specially designed Nordenson camera has now rendered possible the most beautiful and accurate records of retinal abnormalities and disease, and these have of course the virtue of indisputable comparability.

*Prosthesis.*

Prosthesis work has now reached a very high standard. The examples made by a well-known optical firm in London are perfectly soft and plastic, yet they contain no rubber, and once the original cast has been made further copies can be ordered at any time in the sure knowledge of perfect fit, colouring and invisibility. In ophthalmic surgery they are mainly used after exenteration operations for neoplasms, when the eyelids have been removed and the whole orbit cleared out.

*Spectacles.*

The problem of glare has now resulted in the invention of polarising spectacles. Unlike ordinary tinted glasses they do not exert their effect by cutting down the light intensity but, under ordinary conditions, actually polarise the light. This action is of practical importance since most (though not all) glare in nature consists largely of light which has become plane-polarised by reflection; and it is this discomforting part of the light which can be re-polarised and thus eliminated. Salmon fishermen will be interested to know that, thus equipped, it is possible even through the most glinting water to get a clear view of the fish they want to take home.

The trials of prolonged recumbency have now been catered for at the request of the orthopædic surgeons. You may henceforth lie supine in your plaster jacket and comfortably read your book while gazing towards the ceiling or the sky as it may happily be. This is achieved by prismatic bed-spectacles which allow the book to be held on the chest at an angle of 90° from the direction of the eyes.

I scarcely like to end on a morbid note but think you would like to see gas-mask spectacles. They are of importance in that ordinary spectacles are not allowed (for safety's sake) to be worn with the civilian respirator. An attachment is required for fixing special glasses on the outside of the mask. In the case of the Service and Duty types of respirator glasses may be worn inside, but here again they must be of a special design with flat side pieces.

